# Mycelium-Bound Lipase from a Locally Isolated Strain of *Geotrichum candidum*

**DOI:** 10.3390/molecules19068556

**Published:** 2014-06-23

**Authors:** Joo Ling Loo, Anahita Khoramnia, Oi Ming Lai, Kamariah Long, Hasanah Mohd Ghazali

**Affiliations:** 1 Department of Food Science, Faculty of Food Science and Technology, University Putra Malaysia, 43400 Serdang, Malaysia; E-Mails: loojl@utar.edu.my (J.L.L.); khoramnia.anahita@gmail.com (A.K.); 2 Department of Bioprocess Technology, Faculty of Biotechnology and Biomolecular Sciences, University Putra Malaysia, 43400 Serdang, Malaysia; E-Mail: omlai@upm.edu.my; 3 Biotechnology Division, Malaysia Agricultural Research and Development Institute, P.O. Box 12301, 50774 Kuala Lumpur, Malaysia; E-Mail: amai@mardi.gov.my

**Keywords:** mycelium-bound lipase, lipolytic activity, substrate selectivity, *Geotrichum candidum*

## Abstract

Mycelium-bound lipase (MBL), from a locally isolated *Geotrichum candidum* strain, was produced and characterized as a natural immobilized lipase. A time course study of its lipolytic activity in 1 L liquid broth revealed the maximum MBL activity at 4 h for mycelium cells harvested after 54 h. The yield and specific activity of MBL were 3.87 g/L dry weight and 508.33 U/g protein, respectively, while less than 0.2 U/mL lipase activity was detected in the culture supernatant. Prolonged incubation caused release of the bound lipase into the growth medium. The growth pattern of *G. candidum*, and production and properties of MBL were not affected by the scale. The stability of mycelia harboring lipase (MBL), harvested and lyophilized after 54 h, studied at 4 °C depicted a loss of 4.3% and 30% in MBL activity after 1 and 8 months, while the activity of free lipase was totally lost after 14 days of storage. The MBL from *G. candidum* displayed high substrate selectivity for unsaturated fatty acids containing a *cis*-9 double bond, even in crude form. This unique specificity of MBL could be a direct, simple and inexpensive way in the fats and oil industry for the selective hydrolysis or transesterification of *cis*-9 fatty acid residues in natural triacylglycerols.

## 1. Introduction

The current demands of the world’s biotechnological industries are enhancement in enzyme productivity and increasing their shelf life. Enzyme immobilization provides an excellent basis for increasing the availability of enzyme to the substrate with greater turnover over a considerable period of time [1]. Generally, enzymes associated with the mycelia are referred to as mycelium-bound enzymes. Mycelium-bound enzymes could be considered as naturally immobilised enzymes that may be used without the laborious operations of isolation, purification and addition of co-factors, *etc.* Dry mycelium of *Rhizopus oryzae* as immobilized biocatalyst has been successfully applied for ester production [2]. the reactivity and stability of mycelium-bound carboxylesterase from lyophilized cells of *Aspergillus oryzea* has also been explored in organic solvents [3].

Naturally immobilised lipase has many advantages as the free enzyme has low recovery after man-induced immobilisation [4]. Thus, naturally immobilised enzymes have an obvious cost attraction in comparison with the use of free or soluble enzymes in biotransformations of lipids. Hence, fungal mycelia may be used as a direct source of the enzyme thereby eliminating the need for isolation, extraction, purification and external immobilization procedures in cases where the lipases are naturally bound to the cell wall.

In earlier reports, whole cells that contained mycelium-bound lipases have been successfully employed for extracellular lipases produced by fungal species like Aspergillus niger MYA 135 [5]. In view of the many advantages offered by natural immobilised enzymes, some works have been conducted by this laboratory using naturally immobilised lipases. Those studies include the production and properties of Mycelium-bound lipase (MBL) from *G. candidum* [6], *A. flavus* [7,8] and *R. miehei* [9] and their applications in the modification (acidolysis) of several vegetable oils [10], and transesterification of palm kernel olein and anhydrous milk fat mixtures for the preparation of ice cream emulsions [11].

Lipases from fungi are important in industrial applications, especially in enzymatic modification of fats and oils that may involve hydrolysis, transesterification and interesterification processes. Many reports have been published and confirm that the lipase from *G. candidum* is highly specific for monounsaturated *cis*-9 glycerol esters [12,13]. Some researchers classified it as a 1,3-specific lipase that hydrolyses ester bonds where the FA has double bonds, such as oleic acid and linoleic acid [14]. Since palmitic and stearic acids are among the most plentiful saturated fatty acids in common fats and oils, a lipase which would preferentially react with unsaturated fatty acids in the presence of these fatty acids, could be of commercial use for the splitting and restructuring natural TAGs. Additionally, the cost of a process could be reduced by using mycelium-bound or naturally immobilised lipases where repeat usage is possible. Therefore it is also our intention to further explore the potential development naturally immobilised lipases, although the commercial value of such lipases relies on their longevity in use, its physical and catalytic activity.

Earlier studies revealed that *G. candidum* is capable of producing a lipase that hydrolysed up to 70% palm olein during growth [15]. Therefore, lipid hydrolysis using fatty acid-specific lipases could be a very simple and inexpensive means for the production of unsaturated fatty acids. Therefore, the following study was conducted to produce a naturally immobilised lipase (mycelium-bound lipase) from *G. candidum*, isolated earlier from local soil, and study its properties and the potential applications of the enzyme produced.

## 2. Results and Discussion

### 2.1. Effect of Cultivation Scale and Time Course on MBL Activity

Previously it was indicated that the maximum yield of FFA, lipase activity and cell mass during growth of the *G. candidum* strain under study in the presence of palm olein were achieved at different incubation times, *i.e.*, 48, 54 and 96 h, respectively [15]. Hence, a time course study of the lipolytic activity was carried out to confirm these results when the organism was grown in 1 L liquid broth. In addition, mass production of fungal mycelia was carried out batch-wise to induce lipase production. Subsequently, the lipase activity of lyophilized powdered of *G. candidum* mycelia (MBL) was determined.

**Figure 1:**
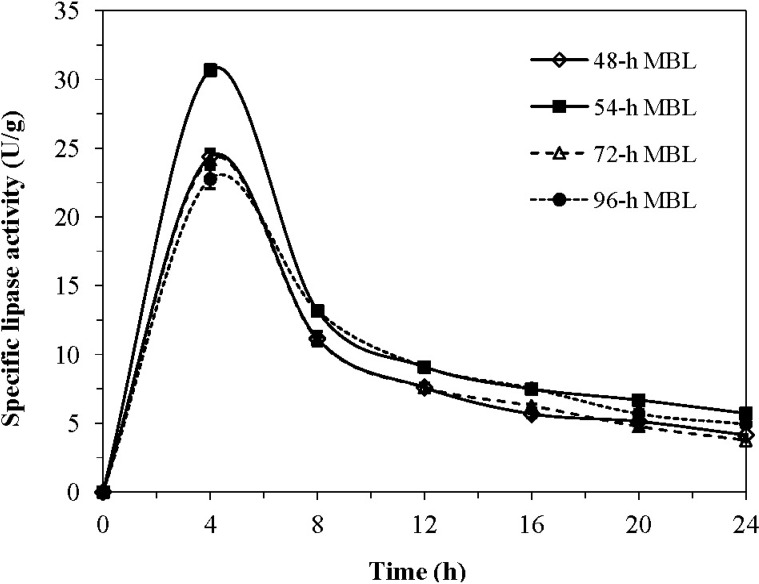
Activity profile as a function of time of MBL harvested at different growth intervals of *G. candidum*. Cultivation was performed at 30°C in 1 L shaken broth inoculated with 2% (v/v) 24-h seed culture and 2% (w/v) sterilised palm olein.

As shown in Figure 1, all MBL investigated displayed a similar trend of a dramatic increase in lipase activity up to 4 h, followed by sharp decrease for another 4 h, and then a gradual decline throughout the rest of the incubation time. Maximum lipase activity was detected in MBL harvested after 54 h of growth and coincident with the highest lipase activity when the organism was cultivated at a smaller scale (100 mL).

MBL harvested earlier at 48 h or later in the growth stage showed lower lipase activity. The reduction in lipolytic activity could be due to release of the bound lipase into the growth medium with prolonged incubation time. This observation was in tandem with those by Jacobsen et al. [16] who reported higher cell-bound activity in *G. candidum* during the early phase of growth and a delay in the secretion of extracellular lipase (after 3 days of growth). Therefore, for subsequent batch-wise mass production of MBL, all mycelium cells were harvested after 54 h of growth at the peak of their lipase activity.

### 2.2. Characteristics and Properties of MBL

Table 1 shows some properties and characteristics of the MBL from *G. candidum* in terms of yield, moisture content, protein content, lipase activity and specific lipase activity. Generally, the yield of MBL obtained ranged from 2.60 to 4.97 g/L dry weight with an average value of 3.87 g/L. In a similar experiment by Jacobsen *et al.* [16] on cultivation of *G. candidum*, maximum dry weight of 5.9 g/L was achieved after 22 h of growth in 800 mL culture medium inoculated with 20 mL or 2.5% (v/v) of spore suspension (about 107 spores/mL). The moisture content in the bound lipase was about 3.85% after 48 h of freeze drying, which was close to the moisture content for Lipozyme (2%–3%). Presence of minimal bound water in the enzyme is crucial for the activation of biocatalyst at the oil-water interface [17]. Lipase activity and specific lipase activity on the average were 22.59 U/g mycelia and 508.33 U/g protein, respectively. The specific lipase activity was relatively low as compared to the purified lipase produced by some of *G. candidum* strains report by Tsujisaka *et al.* [18], which possessed 447 U/mg protein of lipase specific activity.

**Table 1:** Properties and characteristics of MBL from *G. candidum*.

Replicate	Yield (g/L Dry Weight)	* Moisture Content (%)	* Protein Content (mg/g)	* Lipase Activity (U/g)	* Specific Lipase Activity (U/g Protein)
1	2.6	3.7 ± 0.0 ^a^	28.2 ± 1.8 ^a^	16.5 ± 0.6 ^a^	582.6 ± 35.8 ^d^
2	3.0	3.9 ± 0.0 ^bc^	38.8 ± 1.2 ^a^	17.5 ± 1.1 ^a^	450.7 ± 29.1 ^ad^
3	4.6	4.0 ± 0.0 ^c^	39.2 ± 3.9 ^a^	15.7 ± 0.2 ^a^	400.6 ± 29.2 ^a^
4	5.0	3.8 ± 0.1 ^b^	46.0 ± 1.8 ^a^	22.5 ± 1.2 ^b^	489.1 ± 23.1 ^bc^
5	2.3	4.0 ± 0.0 ^c^	55.4 ± 1.4 ^a^	30.4 ± 0.5 ^c^	548.2 ± 4.6 ^cd^
6	4.4	3.8 ± 0.0 ^b^	57.0 ± 1.4 ^b^	33.0 ± 2.5 ^c^	578.8 ± 8.6 ^d^
Mean	3.9 ± 1.1	3.8 ± 0.1	44.1 ± 11.0	22.6 ± 7.5	508.3 ± 74.0

Note: The *G. candidum* culture was grown in 1 L nutrient broth inoculated with 2% (v/v) of 24-h seed culture, 2% (w/v) sterilized palm olein, incubated at 30 °C for 54 h with shaking at 150 rpm. * Results from average of at least trireplicates, totally 18 replicates. Means for the determined values in the same column with different superscript letters denotes significant difference at *p* < 0.05.

A comparison of the lipase activity of MBL indicated lower activity as compared with other commercial fungal lipases such as Lipozyme and Lipase A (96.26 and 50.84, respectively). This is not surprising as both Lipozyme and Lipase A are highly purified commercial preparations of immobilised lipases.

### 2.3. Effect of Storage at 4 °C on the Activity of Mycelium-Bound Lipase

It is generally known that the activity of enzymes may decrease over time during storage. In order to investigate the effect of storage on the activity of the MBL, some lipases were kept in amber bottles flushed with nitrogen gas in a desiccator placed in cold room (4 °C). The activity of the MBL was checked at intervals and the storage stability results are reported in Table 2. There was a loss of 4.3% activity after 1 month of storage. Longer storage times significantly affected the activity of the bound lipase. After 8 months of storage, the lipase retained only 70% of its initial activity. Additionally, it was noticed that the specific activity of the lipase decreased in a similar pattern, showing a 5.67% loss after one month and 25.45% after 8 months of storage, although the protein content remained quite constant. The results proved that the lipolytic activity of MBL deteriorated with storage time. In view of the decreased activity of MBL and also the decline in viability of live culture with prolonged storage, large scale cultivation of cell culture was carried out in batchwise mode and only when required. For a particular experiment, only MBL produced from the same batch of mass culture was used to minimize experimental errors. Thus, it was important to produce the MBL just-in-time when required in order to utilize it while it was still fresh at its optimal activity point. Stability of bound lipase during storage has been reported by [8], who observed no loss in activity of MBL from *A. flavus* during storage for 30 days at 4 °C, but the activity of extracted lipase decreased sharply and its activity was totally lost after 14 days of storage. The stability of MBL as compared to free lipase was probably due to the enhancement of enzyme stability by attachment onto the cell wall that provides some protection to the enzymes.

**Table 2:** Effect of storage at 4 °C on the activity of mycelium-bound lipase.

Storage Time (Month)	Lipase Activity ^a^ (U/g)	Remaining Activity (%)	Specific Lipase Activity ^a^ (U/g)	Remaining Specific Activity (%)
0	20.8 ± 0.4	100.0	550.7 ± 3.7	100.0
1	19.2 ± 0.4	95.7	519.5 ± 3.6	94.3
2	18.1 ± 0.3	88.9	470.5 ± 8.6	85.4
4	17.0 ± 0.5	83.4	447.5 ± 3.2	81.3
8	14.6 ± 0.4	70.0	410.5 ± 3.8	74.6

^a^ Results obtained from average of triplicates, totally 18 replicates of mass culture.

### 2.4. Characteristics and Properties of Free or Extracellular Lipase

The detected free or extracellular lipase activity in the spent culture medium was quite minimal as compared to that of bound lipase. The free lipase activity for all batches of culture medium was below 0.2 U/mL (Table 3). The existing of free lipase in the culture medium would be due to partly release of intracellular or bound lipase through the cell wall membrane during growth. Detection of minimal extracellular lipase activity was desirable as more lipase was deemed intact within the cell wall of the mycelium, with minimal release of the bound lipase into the culture medium. In a study by Charton and Macrea [12] on growth of *G. candidum* CMICC 335426 and its lipase production, they detected lipase activity of 2 U/mL in the culture filtrate, 8 h after inoculation at the start of growth, followed by an increase to 120 U/mL at later stages of the growth. Their observations confirmed that lipase activity is associated with biomass during the early stages of growth, whereas at the end of the growth phase, the activity is found predominantly in the culture filtrate. On the other hand, the release of bound lipase into the culture medium was found to be dependent on the composition, pH and age of the mycelia [16]. However, the release of lipase into the culture broth may not be due to true enzyme secretion instead might originate from cell lysis as observed by Hiol *et al.* [19] during the cultivation of a strain of *R. oryzae*. Many researchers found that addition of surfactants such as Tween 80 stimulated the secretion of hydrolytic enzymes into the culture medium [8,20]. Therefore no surfactant was used during the cultivation of *G. candidum* throughout this study. However, a substantial specific lipase activity was observed, which could be attributed by the high protein content detected in the spent culture medium. This phenomenon was probably due to release of proteinases in the extracellular medium. It may also be due to the accumulation of cell debris after senescence of the mycelia [12]. The use of Tween 80 as the main carbon source resulted in sufficient cell growth and general improvement of the hydrolytic activity toward 2-octylbutyrate in comparison with cells grown on glucose [21]. Also, Tween 80 found to be the most effective inducer for the intracellular lipolytic activity involved in geranyl acetate formation [22].

**Table 3:** Properties of free or extracellular lipase.

Replicate	Lipase Activity (U/mL)	Protein Content (μg/mL)	Specific Lipase Activity (U/g protein)
1	0.1 ± 0.0 ^c^	39.3 ± 4.8 ^b^	2812.7 ± 195.1 ^a^
2	0.1 ± 0.0 ^a^	26.1 ± 0.8 ^a^	2981.8 ± 214.1 ^a^
3	0.2 ± 0.0 ^d^	55.6 ± 1.7 ^c^	3072.9 ± 135.2 ^ab^
4	0.1 ± 0.0 ^c^	28.7 ± 3.5 ^a^	3739.5 ± 306.0 ^c^
5	0.1 ± 0.0 ^b^	23.7 ± 3.0 ^a^	3933.7 ± 174.4 ^d^
6	0.2 ± 0.1 ^d^	44.5 ± 3.4 ^b^	3318.3 ± 235.4 ^bc^
Mean	0.1 ± 0.0	36.3 ± 12.4	3309.8 ± 443.8

Results are the average of at least triplicates, with 18 replicates in total. Means for the determined values in the same column with different superscript letters denote a significant difference at *p* < 0.05.

### 2.5. Properties and Composition of Extracted Lipid Materials

TLC is a fast, simple, easy and useful qualitative method for the detection and separation of lipid classes. Complete separation of the major lipid classes was obtained in a single solvent development in one dimension. The composition of the lipid materials released from 2% (w/v) palm olein was analysed as hydrolysis occurred during mass cultivation of *G. candidum*. Results reveal that the lipid materials hydrolyse into a mixture of MAG, 1,2-DAG, 1,3-DAG, FFA and TAG. The mixture was separated into two fractions after the neutralisation and extraction process; fraction 1 comprised TAG and partial acylglycerols, while fraction 2 was comprised mainly of FFA. The results suggested that the liquid-liquid separation technique employed was effective in separating FFA from a mixture of hydrolysates.

A high temperature gas chromatography (HTGC) method was developed to separate and quantify the lipid classes. Trimethylsilyl (TMS) esters of FA were prepared by exposing the dry acid mixture to a silylating reagent such as hexamethyldisilazane and trimethylchlorosilane or bistrimethylsilylacetamide. These esters are readily formed from the FFA in the presence of other FA esters. Therefore, the products of hydrolysis can be analysed directly, omitting the tedious separation and extraction procedures. The temperature profile of the oven was optimized in order to obtain a baseline separation of the different lipid classes. Peaks were identified based on the retention time of standard molecules (C_16:0_, palmitic acid; C_18:1_, oleic acid; C_18:2_, linoleic acid; C_18:3_, linolenic acid; 1-MP, 1-monopalmitin; 1-MS, 1-monostearin; 1-MO, 1-monoolein; 1,3-DP, 1,3-dipalmitin; 1,3-DS, 1,3-distearin; 1,3-DL, 1,3-dilinoleic; 1,3-DO, 1,3-dioleic; IS, internal standard *i.e.*, cholesteryl stearate; POP, 1,3-dipalmitin-2-olein; POL, 1-palmitin-2-olein-3-linolein; PLL, 1-palmitin-2,3-dilinolein). Upon optimization of the procedure the separation of two critical pairs, oleic and linoleic acids (18:1/18:2), was not established because within the tested temperature profile, co-elution of oleic-linoleic and oleate-linoleate esters was always found. Where peaks corresponding to oleic and linoleic or olein and linolein were superimposed, the total content of both was taken into account. FFAs were always eluted first, followed by MAG, DAG and TAG. Generally components with shorter carbon chains and higher unsaturation eluted earlier than components with longer carbon chains and higher saturation. Repeatability of the described HTGC method was evaluated by analysing at least two duplicates of each oil sample.

The ability of an enzyme to hydrolyse a substrate is reflected by the rate of hydrolysis of the substrate and production of end products [20]. In the present study, the ability of MBL from *G. candidum* to hydrolyse palm olein (substrate) to produce FFA, in particular oleic acid, was studied by analysing the lipid materials extracted from the mass culture.

Table 4 shows the relative percentage of each lipid class present in the extracted lipid materials. The results show that approximately 62% of TAG was hydrolysed to yield 38% of FFA, 22% of DAG and a small amount of MAG (2%).

**Table 4:** Compositions of lipid materials extracted ^a^.

Replicate	FFA (%)	MAG (%)	DAG (%)	TAG (%)
**1**	48.6 ± 1.5	1.5 ± 0.0	19.6 ± 0.6	30.3 ± 1.0
**2**	40.8 ± 2.0	2.0 ± 0.1	23.1 ± 1.1	34.1 ± 3.2
**3**	46.4 ± 0.5	3.1 ± 0.1	23.4 ± 0.6	27.1 ± 0.0
**4**	26.4 ± 0.5	1.1 ± 0.1	25.2 ± 2.0	47.3 ± 2.4
**5**	36.5 ± 1.2	2.9 ± 1.0	18.0 ± 0.9	43.7 ± 0.7
**6**	27.8 ± 0.4	1.3 ± 0.1	24.8 ± 0.7	46.1 ± 0.2
**Mean**	37.7 ± 9.3	2.0 ± 0.8	22.2 ± 3.2	38.1 ± 8.7

^a^ Analysis was carried out in duplicates totally 12 replicates by HTGC method and values expressed as percent weight.

**Figure 2:**
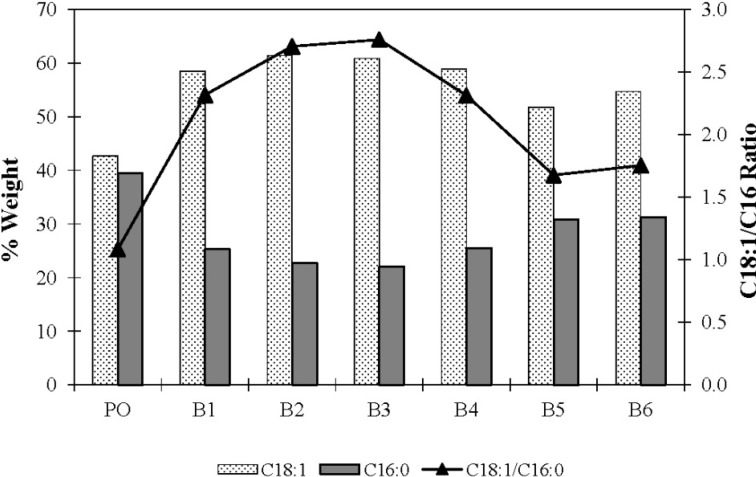
Composition of oleic acid and palmitic acid in starting material, palm olein and FFA fractions extracted from hydrolysates. PO: palm olein; B1-B6: mass culture of batch 1 to 6.

Table 5 shows the compositions of the FFA fraction extracted from the lipid materials and analysed using GC after derivatisation using 14% boron trifluoride-methanol solution. It appears that the composition of the FA released from palm olein by the crude MBL did not differ much in terms of linoleic, linolenic and behenic acid. However, the levels of palmitic and oleic acids analysed in the lipid materials differed markedly from starting material. The FA released from palm olein contained relatively high levels of oleic acid at 57.7% (>42.7%), while in contrast the palmitic acid level was relatively low at 26.3% (<39.43%), suggesting that MBL of *G. candidum* showed selectivity for oleic acid. However, the long chain- saturated fatty acid such as behenic acid (C20) and PUFA such as linolenic acid, was not hydrolysed, indicating that MBL of *G. candidum* does not show specificity or substrate preference for PUFA and long-chain FA. These results are in agreement with those published by other researchers such as Ghazali [6], Jacobsen *et al.* [16]; Baillargeon and McCarthy [23]; Charton and Macrea [12] and Shimada *et al.* [14]. In order to determine the relative rate of hydrolysis of the FA by *G. candidum* lipase, an oleic to palmitic (C18:1:C16) acid ratio was determined. In all cases, the ratio was higher in comparison to palm olein before hydrolysis (Figure 2). Thus, it was evident that substrate specificity and selectivity towards oleic acid had occurred.

**Table 5:** Composition of FFA fractionated from lipid materials following neutralisation and fractionation procedure ^a^.

Replicate	Relative Amount (% Weight)					
	C_16_	C_18_	C_18:1_	C_18:2_	C_18:3_	C_20_
**^b^ PO**	39.4	3.5	42.7	11.8	0.3	0.2
**1**	25.3	1.7	58.5	12.4	0.3	0.3
**2**	22.7	2.1	61.4	10.1	0.5	0.2
**3**	22.1	2.2	60.9	12.6	0.4	0.2
**4**	25.5	2.1	58.9	11.9	0.3	0.2
**5**	30.9	1.9	51.8	11.2	0.2	0.1
**6**	31.2	1.7	54.7	11.3	0.2	0.1
**Mean**	26.3 ± 3.9	2.0 ± 0.2	57.7 ± 3.8	11.6 ± 0.9	0.3 ± 0.1	0.2 ± 0.1

^a^ FFA content was determined by GC following reflux in 14% boron-trifluoride-methanol solution; ^b^ Composition of FA in palm olein as starting material and when 100% hydrolysis occurs. FA was determined by GC-FAME method after derivertisation with sodium methoxide. Values were obtained from 2 duplicates.

## 3. Experimental

### 3.1. Materials

The local *G. candidum* strain (IMI 387428 identified and tagged by the CABI Bioscience Centre, Egham, UK) used was isolated from soil and maintained on yeast-malt extract agar (YMA) slant in the laboratory as described by Loo *et al.* [15]. Seed culture was prepared by inoculating 20 mL of sterilized liquid broth containing 0.5% yeast extract, 1% peptone, and 1% glucose, and was grown for 24 h at 30 °C. Lipozyme RM IM60 from *R. miehei* (immobilised on phenolic type macroporous anion-exchange resin with a particle diameter of 0.2–0.6 μm, moisture content of 2%–3% and lipase activity of 5–6 BAUN/g (Batch Acidolysis, Units Novo)) was purchased from Novozymes (Bagsvaerd, Denmark). Lipase A from *A. niger* (lyophilised lipase powder, lipase activity about 120,000 U/g at pH 6.0) was purchased from Amano Enzyme (Nagoya, Japan). Microbiological media were purchased from Difco (Michigan, MI, USA) and Oxoid (Hampshire, UK). Palm olein was purchased from a local hypermarket in Sri Serdang, Selangor, Malaysia. All chemical reagents and organic solvents used in this study were standard of analytical or HPLC grade from Merck (Darmstadt, Germany) and Fisher Scientific (Leicestershire, UK).

### 3.2. Preparation of Inoculum

The *G. candidum* strain used was a local strain isolated from soil and maintained on Yeast Malt Agar slant with monthly subculture. In order to prepare the inoculum, a loopful of a 24-h culture of *G. candidum* on YMA slant was inoculated into 5 × 20 mL of sterilised liquid broth containing 0.5% yeast extract, 1% peptone and 1% glucose, at pH 7.2, grown for 24 h at 30 °C and was then used as the seed culture for cultivation of *G. candidum*.

### 3.3. Production of Mycelium-Bound Lipase

A growth medium comprising 0.1% peptone, 0.2% glucose, 2% yeast-extract, 0.1% dipotassium hydrogen phosphate and 0.5% ammonium sulphate [15] was dispensed at 1000 mL quantity into five 2000-mL Erlenmeyer flasks, inoculated with 2% v/v of 24 h seed culture, followed by addition of 2% w/v sterilised palm olein. The culture flasks were incubated at 30 °C under 150 rpm shaking in an orbital shaker (Yih Der, LM-510, Yih Der Instrument Co. Ltd, Jhonghe City, Taipei, Taiwan) for up to 96 h. Mycelia were harvested at specified times by a series of filtration through a Whatman No. 4 filter paper fitted in a Buchner funnel. Since soap stocks formed in the culture medium during incubation sometimes impeded the filtration process, washing was also done consecutively with 50 mL hexane and 0.1 M phosphate buffer. The filtrate was then centrifuged (Beckman, J2-21M/E Centrifuge, Palo Alto, CA, USA) at 16,700 *× g*, 4 °C for 15 min to recover the remaining cell mass from the culture. The supernatant was kept at 4 °C for extraction of lipid materials and free or extracellular lipases. The mycelial pellet was collected and washed twice with 30 mL cold 0.1 M phosphate buffer, pH 7.2.

### 3.4. Lyophilisation of Mycelium-Bound Lipase

Mycelium-bound lipase (MBL) was prepared by lyophilizing the mycelia obtained above for 48 h using a shell freeze bench-top system (FreezeZone^®^ 4.5, Labconco, Kansas City, MO, USA). The resulting dry mass was ground into powder using mortar and pestle and stored at 2–3 °C. This lyophilized powder was used as a source of MBL for lipase assay.

### 3.5. Determination of Moisture Content

The analysis was performed with Karl-Fischer volumetric titrator (787 KF Titrino, Metrohm Ltd, Herisau, Switzerland) equipped with a 703 Ti Stand. The solvent system comprised formamide: methanol (7:3, v/v) and CombiTitrant 5 (Merck, Darmstadt, Germany) was used as the titrant. Extraction time was set for 5 min and sample weight was 0.04–0.05 g. Thirty mL of formamide-methanol mixture was added to the titration vessel and titrated to dryness. The lipase sample was then added and titrated in the same manner. Analysis was performed in triplicates.

### 3.6. Lipase Activity Assay

The activity of MBL was determined according to Liew *et al.* [9] by incubating 10 mL of 10% (w/v) palm olein in *n*-hexane as substrate and 2 mL 0.1 M phosphate buffer, pH 7.2 at 30 °C for 1 h with shaking at 120 rpm in a reciprocal water bath (Certomat^®^ W/R, B. Braun, Melsungen, Germany). The enzymatic reaction was initiated by adding 0.1 g of lipase powder or 10 mL of spent culture broth to the reaction medium. Controls were carried out concurrently using inactivated lipase obtained by first sonicating the mycelium for 5 min to disintegrate the mycelia cell prior to boiling for 15 min while free or extracellular lipase was inactivation by boiling only. The reaction was terminated by adding 20 mL ethanol-acetone (1:1, v/v) mixture. The total amount of FFA liberated was titrated against 0.10 N NaOH [24]. One unit of lipase activity was defined as 1 μmole of FA liberated per minute under the conditions used. The specific enzyme activity of MBL was expressed as μmole FFA produced per min per gram protein of dry mycelia powder (U/g protein mycelia). The specific activity of free or extracellular lipase was expressed as μmole FFA produced per min per gram protein of supernatant (U/g protein of supernatant).

### 3.7. Protein Determination

Bradford method [25] was employed to determine the amount of protein using Bio-Rad protein assay kit (Bio-Rad Laboratories, Berkeley, CA, USA). Bovine serum albumin was used as the protein standard. The sample protein content was expressed as gram protein per gram mycelia or gram protein per mL supernatant.

### 3.8. Determination of MBL Activity towards RBD Palm Olein (Lipolysis)

The lipase-catalysed hydrolysis of palm olein was carried out in 50-mL Erlenmeyer flasks at 30 °C and 120 rpm over a period of 24 h. Five grams of RBD palm olein, 5 mL *n*-hexane and 4 mL of 0.1 M phosphate buffer (pH 7.2) were mixed and equilibrated at the reaction temperature. Hydrolysis was initiated by the addition of 0.1 g lipase and stopped by adding 20 mL ethanol-acetone (1:1) solution. The extent or degree of hydrolysis in the reaction mixture was measured by direct titration with 0.10 N NaOH using phenolphthalein as indicator [24]. The blank consisted essentially of the same components mentioned above, except that a heat-inactivated lipase was used to correct for any background titer. The degree of hydrolysis was expressed as the % of FFA (oleic acid) liberated and was corrected for the presence of the acids in the controls. The calculation was based on the Equation (1) as follows:

% FFA = (V_S_ − V_C_) NM/10 W (1)

where, Vs was volume of NaOH used for test sample; Vc was volume of NaOH used for control; N was exact normality of standardized NaOH used (0.10 N); M was molecular weight of the FFA (C18:1 = 282); and W was weight of oil substrate used (g).

This experiment was also carried out using the commercial Lipozyme IM60 and Lipase A as comparisons. Incubation temperatures were set at 60 °C and 30 °C, respectively for these two lipases. Each experiment was carried out in triplicates.

### 3.9. Extraction of Lipid Materials

Lipid materials in the supernatant were extracted three times with 50 mL of *n*-hexane, and the extract was washed with distilled water and dried with anhydrous sodium sulfate [26]. The solvent was removed under reduced pressure with a rotary evaporator (Rikakikai Co. Ltd., Tokyo, Japan) at 55 °C. The lipid residue was decanted into universal bottles and dried in oven at 60 °C for 3 h to remove residual solvent.

### 3.10. Neutralisation and Fractionation of Free Fatty Acids from Extracted Lipid Materials

The method applied in this study was modified from Wang *et al.* [27] using liquid-liquid separation technique. Approximately 10 g of the above extracted lipid was weighed, and then added with 4 mL ethanol and a few drops of phenolphthalein (1% w/v in 95% *iso*-propanol) in a 250-mL separatory funnel. Mild concentration of NaOH (0.5 N) solution was added gradually to neutralise the FFA. Neutralisation is an exothermic reaction, and to prevent further breaking down of TAG, the solvents used for extraction was chilled. The mixture was then extracted a few times with n-hexane, with gentle shaking of the funnel. Vigorous shaking was avoided to prevent formation of emulsion at the biphasic layer once hexane was added. However, a little chilled ethanol or distilled water was added to break any emulsion that formed. The top organic phase (*n*-hexane), which mainly consisted of MAG, DAG and TAG, was decanted and kept. A 20 μL of this fraction was spotted on TLC plate to check its purity.

The aqueous layer that remained in the separatory funnel was added with a few drops of concentrated hydrochloric acid (HCl, 5.0 N) solution until oil layer appeared on top. This top oil layer was collected and washed a few times with warm water (approximately 60 °C) to rid of NaOH, HCl and phenolphthalein. After washing, the oil fraction was centrifuged at 5000 rpm for 10 min to remove residual water from oil. Anhydrous sodium sulphate was incorporated into the washed oil to further absorb any remaining moisture. This mixture was then filtered through a Whatman No. 4 filter paper and solvent was removed in vacuo. The purity of this FFA fraction extracted was checked by TLC method. Lipid classes were identified by the relative positions of highly pure standards spotted onto the plates.

### 3.11. Determination of Total Free Fatty Acids

The PORIM Test Method [24] was employed to determine the FFA content of the lipid materials extracted from supernatant. The experiment was carried out in duplicate. Ten grams (±0.05 g) of lipid material was weighed into a 250-mL Erlenmeyer flask and 50 mL of neutralized isopropanol was added to the flask. The flask was heated at 40 °C for 5 min and then titrated to a phenolphthalein (1% in 95% isopropanol) end point with 0.10 N NaOH. The amount of FFA was expressed as percent of oleic acid.

### 3.12. Analysis of Fatty Acids Composition

The fatty acid composition of untreated oil samples were analysed using gas liquid chromatography. Methyl esters of fatty acids (FAME) were prepared according to PORIM Test Methods [24] by methanolysis of the glycerides in an alkaline medium. Approximately 50 mg of oil sample was weighed into a 2 mL Eppendorf tube, followed by addition of 0.95 mL of chloroform and 0.05 mL of sodium methoxide. The mixture was vigorously mixed for 5 s with the help of a vortex mixer. The mixture first went clear and then turbid as sodium glyceroxide was precipitated. The clear upper layer of methyl esters were analysed by Shimadzu gas chromatography, model GC-14A (Shimadzu Corporation Kyoto, Japan), equipped with a flame-ionization detector (FID) and a SCL-10A system controller. The stationary phase comprised of a polar capillary column BPX-70 (0.32 mm i.d., 30 m length and 0.25 m film thickness, SGE Australia Pty. Ltd., Ringwood, Australia). Hydrogen gas at flow rate of 0.6 kg/cm^2^ was used as the carrier gas, nitrogen at 1.4 kg/cm^2^ was used as the makeup gas and compressed air was set at a flow rate of 0.5 kg/cm^2^. The temperature of both injector and detector were maintained at 220 °C. The injection mode was splitless and 0.5 μL sample was injected into a 10-μL loop through SIL-10A auto injector. The initial column oven temperature was 80 °C, and increased at a rate of 8 °C/min to 180 °C until the analysis completed. FAME peaks were identified by comparison of retention time to a standard mixture. Quantification of each component was based on scan area normality. All analyses were carried out in triplicates.

### 3.13. Analysis of Free Fatty Acid Composition

This analysis was carried out on the FFA portion neutralised and fractionated from the lipid materials following their extraction. As the conventional method of preparing FAME using alkaline catalyst, sodium methoxide was ineffective as the samples contained high amount of FFA (>5%) [28], the FFAs were methylated under acidic condition by ester exchange with 14% boron triflouride-catalysed transesterification. FAMEs were prepared according to PORIM test methods [24] by transesterification of 50 mg of oil in 14% BF3-methanol solution under reflux condition for 1 h. After reflux, approximately 100 mL of saturated sodium chloride solution and 3 mL of heptane were added. The FAME was collected in heptane layer. These methyl esters were injected into GC as described above. Every sample was analysed in triplicates.

### 3.14. Quantification of Lipid Class

The complex mixtures of FFA, MAG, DAG and TAG in the extracted lipid materials were determined by a high temperature-gas chromatographic (HTGC) method without saponification of the sample as described by Verleyen *et al.* [29]. The oil samples were derivatized in order to increase the volatility of the components. A molten oil sample of 0.05 g was directly weighed into a 2-mL Eppendorf tube. After dissolving the sample in 0.3 mL of pyridine and addition of 0.2 mL *N*,*O*-bis-(trimethyl)trifluoroacetamide (BSTFA) containing 1% trimethylchlorosilane (TMCS) as derivatizating and silylating agent, 50 μL of cholesteryl stearate solution with known concentration (±40 mg/mL pyridine) was added as internal standard. The Eppendorf tube was placed in a 70 °C oven for 30 min for completion of the silylation. This treatment produced trimethylsilyl (TMS) esters and esters of FFA, mono- and DAG, respectively. The sample was then diluted with 0.5 mL of chloroform and ready for injection.

Separation was performed on a Shimadzu 14-A GC equipped with an Equity-5 (15 m × 0.32 mm × 0.25 μm, Supelco, Bellefonte, PA, USA) capillary column with detection by FID. An optimal temperature programme for a good separation of the lipid classes was established as follows: injection at 170 °C, oven heating increased at 10 °C/min to 240 °C with 2 min hold, continued at a speed of 15 °C/min to 340 °C with 3 min hold then at 5 °C/min to 360 °C with 10 min hold. The total programmed time was 33.67 min. The detector temperature was set at 360 °C. Oxygen free-nitrogen was used as the carrier gas at a pressure of 35 kPa. Total flow was set at 25 mL/min, with a velocity of 30 cm/s at split ratio 17:1. Calibration graphs were constructed using pure standards of FFA, MAG, DAG and TAG. Analysis was carried out in triplicates.

## 4. Conclusions

The study shows that a locally isolated *G. candidum* was able to produce mycelium-bound lipase (MBL) when it is grown in the presence of an oil. Once dried, MBL could be used to carry out lipolytic activity in the presence of water. It is anticipated that if water is removed from the reaction medium, MBL can also be used to carry out synthetic reactions such as esterification. Mycelium-bound lipase from *Geotrichum candidum* strain as an economic, naturally immobilized, substrate selective and safe (from GRAS organism) lipolytic enzyme with long time stability at 4 °C would be a valuable biocatalyst in the fat and oil industries.
